# Spatial profiling reveals association between WNT pathway activation and T-cell exclusion in acquired resistance of synovial sarcoma to NY-ESO-1 transgenic T-cell therapy

**DOI:** 10.1136/jitc-2021-004190

**Published:** 2022-03-09

**Authors:** Katie M Campbell, Maneesha Thaker, Egmidio Medina, Anusha Kalbasi, Arun Singh, Antoni Ribas, Theodore Scott Nowicki

**Affiliations:** 1 Medicine, Division of Hematology/Oncology, University of California Los Angeles, Los Angeles, California, USA; 2 Department of Molecular and Medical Pharmacology, University of California, Los Angeles, Los Angeles, California, USA; 3 Radiation Oncology, University of California, Los Angeles, Los Angeles, California, USA; 4 Jonsson Comprehensive Cancer Center, University of California, Los Angeles, Los Angeles, California, USA; 5 Surgery, Division of Surgical Oncology, University of California, Los Angeles, Los Angeles, California, USA; 6 Parker Institute for Cancer Immunotherapy, San Francisco, California, USA; 7 Pediatrics, Division of Pediatric Hematology/Oncology, University of California, Los Angeles, Los Angeles, California, USA; 8 Microbiology, Immunology, & Molecular Genetics, University of California, Los Angeles, Los Angeles, California, USA; 9 Eli and Edythe Broad Center for Regenerative Medicine and Stem Cell Research, University of California, Los Angeles, Los Angeles, California, USA

**Keywords:** immunotherapy, adoptive, sarcoma, tumor escape, immune evation, tumor microenvironment

## Abstract

**Background:**

Genetically engineered T-cell immunotherapies for adoptive cell transfer (ACT) have emerged as a promising form of cancer treatment, but many of these patients develop recurrent disease. Furthermore, delineating mechanisms of resistance may be challenging since the analysis of bulk tumor profiling can be complicated by spatial heterogeneity.

**Methods:**

Tumor samples were collected from a patient with synovial sarcoma who developed acquired resistance to ACT targeting NY-ESO-1. Biopsies (primary, progressive metastasis, and recurrence) were subjected to bulk tumor DNA and RNA sequencing, as well as high-dimensional spatial profiling of RNA and protein targets. Untreated and progressive lesions were compared with identified patterns associated with acquired resistance to ACT.

**Results:**

Gene expression patterns due to immune activity and infiltration were diluted in bulk tumor sequencing. The metastasis was enriched for tumor regions with increased *CTNNB1* (encoding beta-catenin), which were negatively associated with the expression of T-cell surface proteins and antigen presentation machinery. Spatial profiling was most highly concordant with bulk sequencing in the lesions with decreased spatial heterogeneity.

**Conclusions:**

Complementary use of bulk and spatial profiling enables more accurate interrogation of tumor specimens, particularly to address complex questions regarding immunotherapeutic mechanisms. Our study uses this approach to demonstrate a mechanism of T-cell exclusion and resistance to cellular immunotherapy in synovial sarcoma.

## Introduction

Genetically engineered T-cell immunotherapy has emerged as a potent and widely applicable form of cancer treatment. Current approaches use viral vectors to encode a transgenic T-cell receptor (TCR) or chimeric antigen receptor (CAR) in autologous patient T cells, which targets a specific tumor antigen and leads to a robust anticancer response mediated by cytotoxic T-cell infiltration of the tumor. Synovial sarcoma is an aggressive mesenchymal neoplasm with an overall 10-year disease-free survival of 50%, and patients diagnosed with metastases have a median survival of only 7–37 months.^1^

Synovial sarcoma is characterized by the balanced chromosomal translocation t(X,18; p11, q11) resulting in an oncogenic fusion protein, SS18-SSX, in over 90% of all cases.^1–4^ The SS18-SSX protein displaces SMARCB1 from the core SWitch/Sucrose Non-fermentable (SWI/SNF) complex and dysregulates gene expression of the WNT–beta-catenin signaling pathway.^5^ WNT–beta-catenin is increasingly recognized as a major driver for T-cell regulation within the tumor microenvironment and can contribute to acquired resistance to immunotherapies which rely on T-cell infiltration.^6 7^ In addition, the SS18-SSX fusion protein leads to abnormal epigenetic regulation in the synovial sarcoma cell, causing aberrant NY-ESO-1 expression. Due to the consistent and uniform expression of the cancer-testis antigen NY-ESO-1, synovial sarcoma presents an excellent opportunity for adoptive cell transfer (ACT) targeting this antigen.^2–4^

Here we describe a patient with metastatic synovial sarcoma who was treated with serial dosing of transgenic TCR ACT targeting the tumor antigen NY-ESO-1 in combination with dendritic cell vaccination. On disease progression, the patient was treated with an identical cell therapy regimen in combination with the CTLA-4 blocking immune checkpoint antibody ipilimumab. In both instances, the patient had robust antitumor responses to therapy but developed disease progression in under a year.^2^ Since initial studies involving traditional bulk sequencing methodologies were unable to detect any unifying molecular aberrations responsible for these relapses, we used a combination of bulk sequencing and spatial profiling approaches to demonstrate that T-cell infiltration was impaired at the time of disease progression, associated with spatially distinct increases in WNT–beta-catenin expression within the metastatic lesion. These results underscore the need for the development of therapeutic approaches which can overcome these tumor intrinsic mechanisms of resistance to transgenic cellular immunotherapies.

## Methods

### Sample acquisition

The patient was enrolled in NCT02070406 and NCT01697527, as previously described,^2^ for the collection of the primary, metastatic, and recurrent lesions and peripheral blood characterized in this study.

### Nucleic acid sequencing and analysis

Nucleic acid extraction and library preparation was performed by the Technology Center for Genomics and Bioinformatics at University of California, Los Angeles (UCLA). DNA was extracted from formalin-fixed, paraffin-embedded (FFPE) biopsy samples using a Biochain AnaPrep Automated Nucleic Acid Preparation System. DNA was extracted from peripheral blood mononuclear cells (PBMCs) using the Qiagen AllPrep DNA/RNA isolation kit, according to the manufacturer’s protocol. Libraries were generated using NimbleGen SeqCap EZ library preparation per the manufacturer’s protocol and sequenced on the Illumina HiSeq 3000 platform (2×150 bp). Whole-exome sequencing (WES) reads were aligned to the human reference genome (GRCh38) using BWA-MEM v0.7.15.^8^ Duplicates were tagged using Picard MarkDuplicates v2.16.0 (http://broadinstitute.github.io/picard), and reads were recalibrated using GATK4.^9^ Single nucleotide variants (SNV) were called using the union of Mutect2,^10^ Varscan2,^11^ Strelka,^12^ and SomaticSniper,^13^ and small insertions and deletions (indels) were called using Mutect2 and Varscan2. Manual review of SNVs was automated using the DeepSVR algorithm.^14^ SNVs and Indels were filtered to those that were called by at least two variant callers and were not failed as false positives classified by DeepSVR. Copy number and loss-of-heterozygosity (CN/LOH) analysis was performed by Sequenza.^15^

RNA was extracted from FFPE biopsy samples using a Biochain AnaPrep Automated Nucleic Acid Preparation System. mRNA libraries were generated using the Kapa Stranded mRNA Kit, and were subjected to 2×150 bp sequencing on the Illumina HiSeq 3000 platform. RNA sequencing (RNAseq) data was aligned to the human reference genome (GRCh38) using HISAT2,^16^ and gene expression quantification was performed using Stringtie^17^ and the Ensembl reference transcriptome (v96). Gene fusions were identified using kallisto^18^ and pizzly.^19^ Single sample gene set enrichment analysis (ssGSEA) was performed using the GSVA R package^20^ across the KEGG, Reactome, HALLMARK, Regulatory Target Gene Sets, Immunologic signature Gene Sets, and Curated Gene Sets available through the msigdbr R package.^21^

Genomic DNA was isolated and productive TCRβ sequences were identified from formalin-fixed, paraffin-embedded tumor biopsies, patient-matched infusion products, and post-infusion PBMCs, as previously described.^2^

### GeoMx digital spatial profiling (DSP)

For protein and gene expression spatial profiling in the tumor samples, the GeoMx Digital Spatial Profiler (NanoString Technologies) was used, as previously described.^22^ Three 5 um slides were cut from each FFPE tumor block and shipped to NanoString Technologies for processing (Seattle, Washington, USA). Four fluorescent markers were applied to one slide-mounted FFPE tissue section: NY-ESO-1 (Santa Cruz Biotechnology sc-53869), pan-cytokeratin, CD45, and Syto 83 nuclear stain. Images at ×20 magnification were used to identify regions of interest (ROI) either within the tumor bed or at the invasive margin (where the tumor tissue abutted surrounding normal tissue) to include regions with varying degrees of CD45 expression. ROIs were then processed by microscope automation for UV-light cleavage of indexed oligos. With each illumination cycle, photocleaved oligos were collected and hybridized for analysis on the NanoString nCounter Analysis system. Target expression values were normalized by the area and positive control signal (External RNA Controls Consortium [ERCC] reference material). Gene and protein targets were annotated by the hallmark gene sets,^21^ and the most recurrently annotated gene set was chosen as a single annotation for each target.

### Data and statistical analysis

Data analysis was performed using R V.4.0.2, and plotting was carried out using the ggplot2,^23^ ggalluvial,^24^ and patchwork (https://github.com/thomasp85/patchwork) R packages.

## Results

### Clinical course

**Figure 1:**
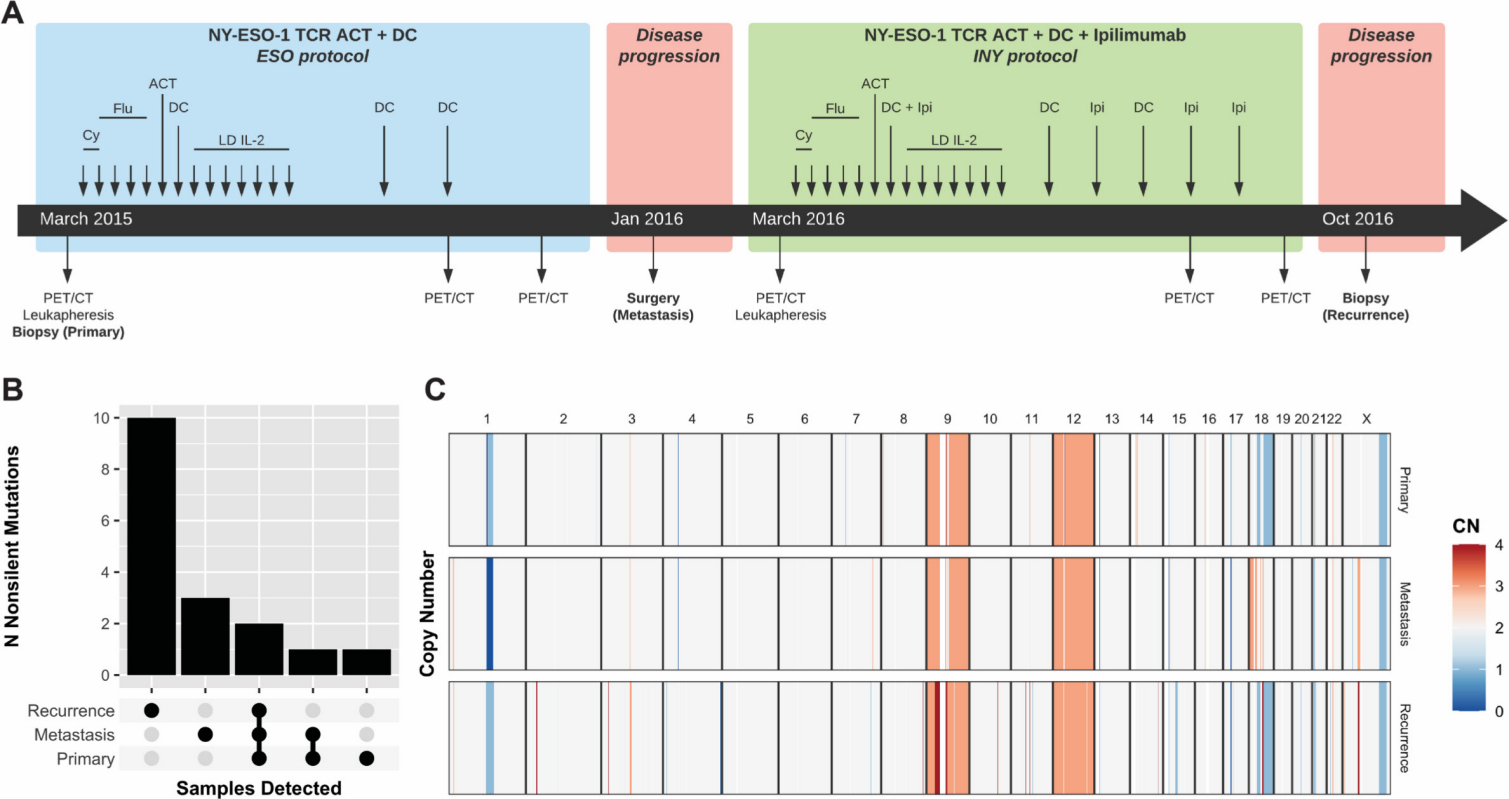
Genomic landscape of the primary, metastatic, and recurrent lesions. (A) Overview of the clinical and sample collection timeline for the analysis performed in this study. (B) The number of non-silent mutations shared within and across samples is shown (see also online supplemental table S2). (C) The copy number landscape is shown, depicting copy number gains (red) and losses (blue), detected by WES, the primary, metastasis, and recurrence samples. ACT, adoptive cell transfer; Cy, cyclophosphamide; CN, copy number; DC, dendritic cell; Flu, fludarabine; IL, interleukin; Ipi, ipilimumab; LD, low-dose; PET, positron emission tomography; TCR, T-cell receptor; WES, whole-exome sequencing.

The patient was a Caucasian woman in her early 40s who initially presented in May 2013 with a monophasic synovial sarcoma in her right popliteal fossa with lung metastases (figure 1A). The tumor was noted to be strongly NY-ESO-1 positive, with little T-cell infiltration by immunohistochemistry (IHC) and T-cell receptor beta (TCRβ) sequencing.^2^ She was refractory to standard-of-care chemotherapy (doxorubicin/ifosfamide) and radiation therapy, so she was enrolled in NCT02070406, in which she was treated with autologous NY-ESO-1-specific TCR transgenic lymphocytes along with NY-ESO-1 peptide-pulsed dendritic cell (DC) vaccination in March 2015.^2^ She tolerated treatment well and demonstrated a partial response in all sites of disease. Subsequent imaging in December 2015 demonstrated disease progression in her lung metastases. One metastasis was selected for surgical resection and demonstrated robust T-cell infiltration and continued NY-ESO-1 expression. The patient was enrolled in NCT01697527 and in March 2016 received autologous transgenic NY-ESO-1 TCR lymphocytes with NY-ESO-1-pulsed DC vaccination, along with the anti-CTLA4 agent ipilimumab 3 mg/kg intravenously every 3 weeks for four total doses.^2^ Her tumor lesions again initially responded to treatment but in September 2016 developed disease progression. Her recurrent primary right popliteal fossa lesion was biopsied in October 2016, again demonstrating NY-ESO-1 expression and T-cell infiltration. She was subsequently treated with nivolumab and pazopanib in succession, both of which failed to stop further disease progression, and the patient passed away in October 2018.

10.1136/jitc-2021-004190.supp1 Supplementary data

### DNA sequencing does not indicate clear genetic drivers of resistance

WES was performed on the three tumor samples and patient-matched normal blood (online supplemental table S1), for somatic variant profiling. Primary, metastatic, and recurrent lesions had 89%, 88%, and 95% tumor cellularity, respectively, and there were 17 non-silent mutations detected across these three timepoints (figure 1B and online supplemental table S2). There were two mutations shared across all three samples (*FLRT3,* P32S; *CACNA2D3*, G358E); three mutations were specifically detected in the metastasis (*ZNF821*, I223M; *GRM7*, N279T; *TTBK1*, A1106V), and 10 were specifically detected in the recurrence, suggesting the outgrowth of a novel subclone in the recurrent lesion. There were no non-silent mutations that were shared by both the metastasis and recurrence and not the primary. All three lesions shared the t(X, 18; p11, q11) *SS18-SSX1* gene fusion, canonically described in synovial sarcoma.^25^ Overall, there were no clear de novo genetic drivers of disease progression in either the metastasis or the recurrence (figure 1B, C).

### Transgenic NY-ESO-1 TCR clones persist over the course of treatment

**Figure 2:**
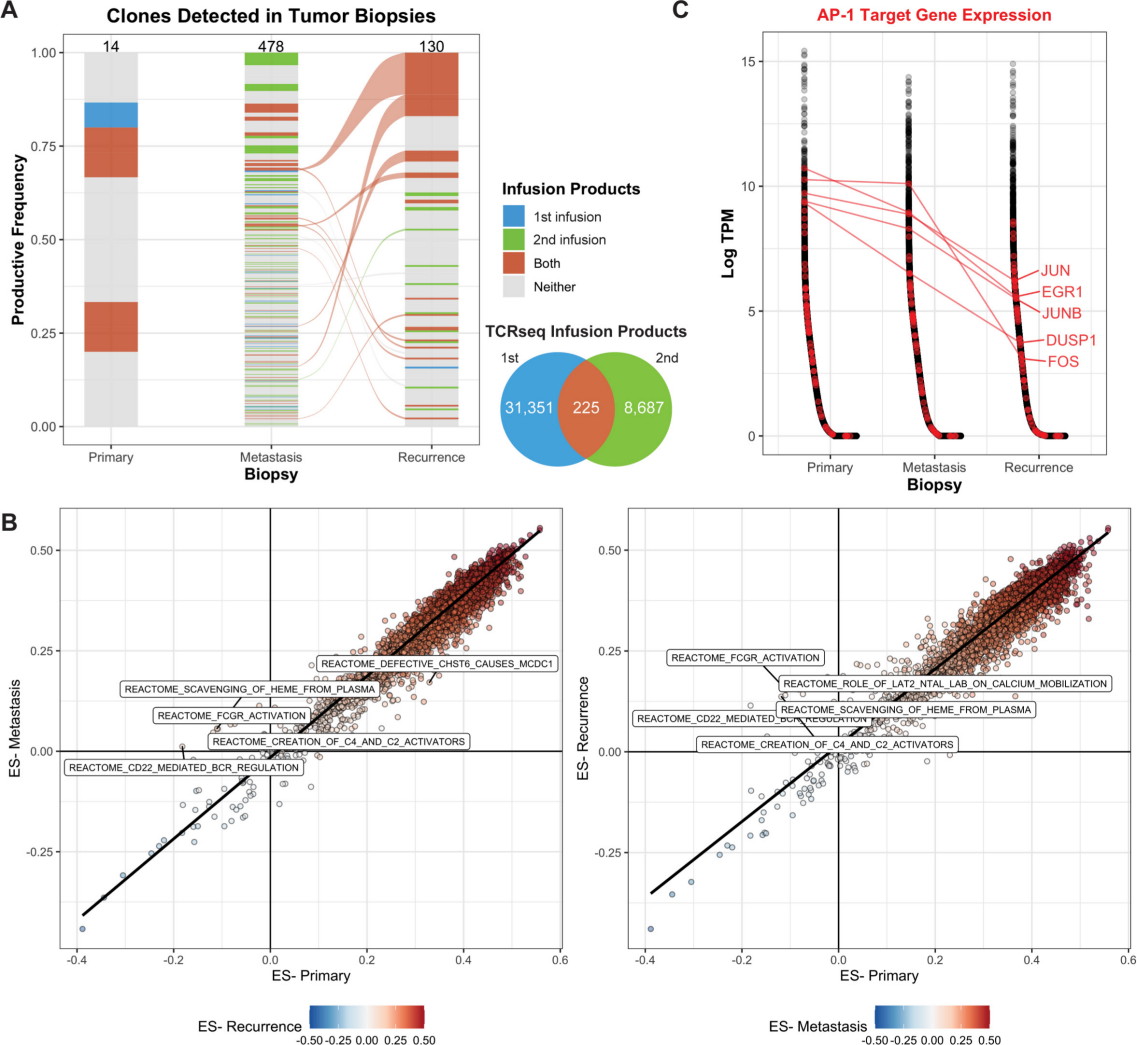
Transgenic TCR products persist over the course of disease progression. (A) TCR clones that were detected in the sorted NY-ESO-1 TCR+ infusion products (either the first, second, or both) were identified in TCRseq derived from bulk tumor DNA. The number of clones detected in each product is indicated by the Venn diagram and by the numbers at the top of each bar. The width of the clone indicates the productive frequency of the TCR repertoire, excluding the NY-ESO-1 TCR, and clones connected between timepoints (alluvium) indicate that it was detected in both the metastasis and recurrent lesions. (B) Single-sample gene set enrichment analysis was used to quantify the ES across annotated gene sets. Each point represents a gene set, comparing either the metastasis (left) or recurrence (right) to the primary lesion. The top five reactome datasets with the highest differences in ES are labeled. (C) Genes (x-axis) were sorted by decreasing expression in log2-transformed transcripts per million (log-TPM, y-axis) within each biopsy transcriptome profiling. Genes associated with the Pathway Interaction Database AP1 pathway (transcription factor network) are labeled in red, and a selected subset is labeled. ES, enrichment score; TCR, T-cell receptor.

Our previous work revealed the presence of the NY-ESO-1 transgenic TCR in both the metastatic and recurrent lesions using T-cell receptor sequencing (TCRseq), indicating that the infusion product successfully trafficked to both sites and remained present following disease progression.^2^ We further interrogated TCRseq data derived from the infusion products, and tumor samples to define the clonal dynamics across the two clinical trial protocols and disease progression. Since the patient underwent two infusions, the endogenous coexpressed native TCR clones detected in either or both sorted (NY-ESO-1 TCR+) infusion products were annotated in TCRseq derived from the tumor biopsies. Of the 14 endogenous coexpressed TCR clones detected in the primary tumor, 5 were detected in the NY-ESO-1 TCR+ infusion products; however, none of these clones were also detected in either the metastasis or recurrence (figure 2A). There were 478 unique clones detected in the metastasis and 130 clones detected in the recurrence lesion; 140 (29.2%) and 25 (19.2%) were clones present in at least one infusion product, comprising a cumulative 35.3% and 33.0% productive frequency of the clones detected.

These data suggest that the ACT products were effectively trafficking and persisting at the tumor sites, so we queried the bulk transcriptome profiling for gene sets or processes that may be differentially expressed across the three biopsies. Overall, the enrichment scores across 14,186 gene sets were highly concordant between the metastasis and primary (Spearman r, 0.938; p<2.2e-16) and the recurrence and primary (Spearman r, 0.954; p<2.2e-16; figure 2B). Gene sets, including Reactome FcgR Activation and Reactome CD22 Mediated BCR Regulation, were more highly enriched in the metastasis and the recurrence than in the primary (figure 2B), suggesting the presence of B cells in the progressive timepoints.

We hypothesized that while the T cells effectively trafficked to the tumor site, their exhaustion or dysfunction may have resulted in tumor outgrowth. Thus, we queried the bulk transcriptome for genes that may be associated with these processes. AP-1 target genes, particularly *JUN*, have been previously implicated in exhaustion of CAR T-cell therapy following tonic CAR signaling.^26^ We identified a subset of genes associated with AP-1 signaling (*JUN*, *EGR1*, *JUNB*, *FOS*, and *DUSP1*) that were expressed at much lower levels in the recurrence, compared with the primary lesion (median 36.2X, 11.5–163.1X; figure 2C). Furthermore, these genes were all expressed at lower levels in the recurrence, compared with the metastasis lesion (median 7.4X, 6.4–146X), suggesting that this pattern was specific to the recurrence lesion.

### T-cell exclusion is associated with tumor CTNNB1 expression

**Figure 3:**
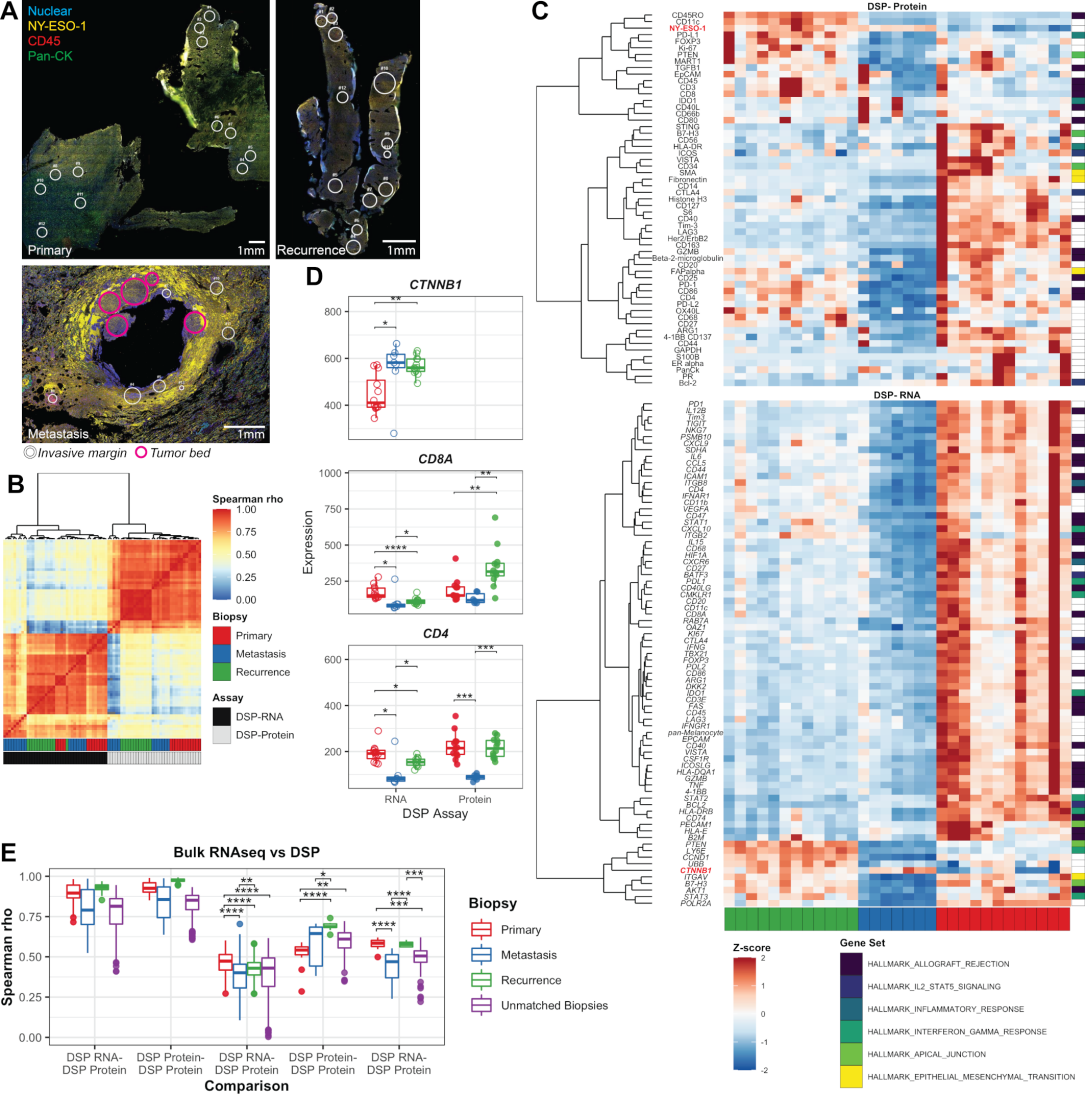
Spatial profiling across samples. (A) Confocal microscopy images: overlaying nuclear staining (blue), NY-ESO-1 (yellow), pan-cytokeratin (green), and CD45 (red) were used to identify ROIs, indicated by circles. Pink circles in the metastasis lesion indicate those that were within the tumor region. (B) Heatmap of the Spearman rank correlation across all matched protein–RNA markers for all ROIs assessed. Each ROI is indicated by the corresponding biopsy and assay by the color bars at the bottom of the plot. (C) Heatmap of the marker-scaled expression of proteins (top) and genes (bottom) assessed across ROIs; non-tumor regions from the metastasis are not included in this figure. Markers were clustered by assay type and scaled expression using unsupervised hierarchical clustering with Ward agglomeration. Markers are also annotated on the right by corresponding hallmark gene sets. (D) Raw expressions of *CTNNB1*, CD8/*CD8A*, and CD4/*CD4* are shown on the y-axis across ROIs derived from each sample (denoted by color). (E) The Spearman rank correlation (y-axis) was quantified across all matched protein or RNA markers across ROIs and between ROIs and matched bulk RNA samples. Comparisons are indicated along the x-axis. (D, E) Pairwise Wilcox tests were performed across groups. Non-significant comparisons are not shown; significant differences are indicated across comparisons. *P<0.05, **P<0.01, ***P<0.001, ****P<0.0001. DSP, digital spatial profiling; ROI, region of interest;

The genomic profiling did not indicate a clear tumor-intrinsic genetic mechanism of resistance in either the metastatic or recurrent lesion, and the conclusions in the bulk transcriptome profiling were limited, since we could not directly associate the downregulation of AP-1 targets to T-cell exhaustion. However, our results did suggest that the infusion product had successfully trafficked to these lesions and that the NY-ESO-1 antigen target was still present in the progressive lesions. We next applied spatial profiling using the Nanostring GeoMx DSP platform to interrogate physical tumor-immune cell interactions to determine whether the T-cell product was able to infiltrate the tumor. Multiplex staining for nuclear, NY-ESO-1, CD45, and pan-cytokeratin were used to select 12 ROIs for each sample (figure 3A). Each sample was profiled for 57 proteins and 78 genes, including 31 matched gene–protein targets (online supplemental table S4).

When ROIs were compared within and across samples, expression profiles generally clustered together based on sample and assay (figure 3B), particularly in the primary and recurrence lesions. Overall, expression of the therapeutically targeted NY-ESO-1 was lower in the regions queried in the primary lesion, compared with the metastatic and recurrent samples, and expression patterns exhibited increased heterogeneity, with varied inflammatory markers. Regions of the metastasis clustered based on their presence within the tumor bed versus the invasive margin (figure 3B), and we identified increased expression of *CTNNB1* within the metastasis tumor bed (figure 3C). Furthermore, these areas also showed little to no expression of markers associated with inflammatory or interferon signaling (including CD45, CD3, CD8, CD4, and PD-L1), indicating that the T cells derived from the infusion product detected by TCR sequencing were not present within the tumor bed. Regions of the recurrence lesion also showed increased expression of *CTNNB1* and tumor marker NY-ESO-1, compared with the primary lesion; however, these regions also exhibited the expression of inflammatory genes, including T-cell markers and checkpoints (CD3, CD8, PD-1, and CD4; figure 3C, D) and markers of other immune cell types (eg, CD20, CD163, and CD56). This further suggested that T-cell and immune cell exclusion was specific to the metastatic lesion.

The markers explored by DSP were compared with gene expression quantified by bulk RNAseq. Overall, the Spearman rank correlation between bulk RNAseq and ROIs evaluated by RNA was higher (figure 3E) than the association between bulk RNAseq and ROIs measured by protein expression (Wilcoxon test, p=4.15e-11). The variance in ROI correlation with RNAseq was greatest in the metastasis (0.018 with DSP RNA, 0.010 with DSP protein) and lowest in the recurrence (6.5e-3 with DSP RNA, 3.1e-4 with DSP protein). This corresponded to the heterogeneity seen in the ROIs from the metastasis wedge resection, where the ROIs within the tumor bed were more highly correlated with the bulk RNAseq (median 0.69, 0.67–0.71 with DSP RNA; median 0.51, 0.47–0.55 with DSP protein) compared with the invasive margin of the tumor (median 0.43, 0.38–0.62 with DSP RNA; median 0.36, 0.24–0.48 with DSP protein).

## Discussion

High uniform expression of the cancer-testis antigen NY-ESO-1 in synovial sarcoma makes it an attractive target for ACT, and this approach has demonstrated objective clinical responses in 61%–67% of patients.^4^ However, these responses are not durable, with patients often experiencing progression of disease within 6–12 months.^4^ To enhance responses and improve outcomes in these patients, it is essential to understand the mechanisms driving treatment resistance/failure to better identify combinatorial or alternative therapies. Our previous work demonstrated persistent antigen expression and persistence of the NY-ESO-1-restricted ACT infusion product in a patient with metastatic disease progression and recurrence after displaying tumor regression. In this study, spatial profiling revealed T-cell exclusion by the tumor, which was associated with increased expression of *CTNNB1* in a metastatic lesion following disease progression. This pattern was not observed in the recurrence at the primary site, indicating diverging mechanisms of progression following ACT. Importantly, these patterns were attenuated in bulk tumor expression profiling and were only apparent when spatial profiling approaches were employed.

Several mechanisms have been identified in primary and acquired resistance to T cell-based immunotherapies in other cancer types, including inadequate T-cell infiltration, loss of T-cell function, and loss of antigen presentation.^27^ T-cell exclusion from the tumor microenvronment has been widely studied in melanoma and is associated with activation of the WNT–beta-catenin signaling pathway.^7 28^ This pathway inhibits the expression of chemokine genes such as *CCL4*, preventing the recruitment of Batf3-lineage dendritic cells which are subsequently unable to prime CD8+ T cells.^7 28^ Studies in melanoma and urothelial bladder cancers have demonstrated WNT–beta-catenin-mediated T-cell exclusion as a mechanism of resistance to immune checkpoint blockade therapies, tumor antigen vaccination, and ACT.^7 29^ In the patient presented in this study, the expression of lineage markers for both T cells (eg, CD3, CD8, and PD-1) and other immune cells (eg, CD20, CD56, and CD163) were anticorrelated with increased expression of *CTNNB1* within the tumor regions. While bulk TCR profiling indicated the presence of the NY-ESO-1-restricted TCR and coexpressed TCR clonotypes identified in the ACT infusion product, spatial profiling suggests that these T cells were located in areas of the tumor with low *CTNNB1* expression levels and were not present in tumor areas with increased *CTNNB1* expression. Furthermore, while the bulk RNA profiling revealed slightly increased expression of B-cell markers and activation, these markers were concordant with the expression of other immune cell types in ROIs queried by spatial profiling and were absent in *CTNNB1* expressing regions in the metastasis. Of note, while our treatment protocols did not allow for DC vaccination beyond the first month post-ACT, it is possible that the DC vaccine might have been able to help prevent the onset of CTNNB1-driven resistance. Indeed, FLT3L-induced bone marrow dendritic cells were able to partially overcome CTNNB1-driven resistance to T-cell infiltration in melanoma.^30^ Future work with longer-term DC vaccination may prove useful in circumventing T-cell exclusion driven by CTNNB1 in ACT.

A major limitation to this study is the impact of sampling approaches on interrogating tumor-immune dynamics. While spatial profiling is advantageous in differentiating the tumor landscape, the identification of T-cell exclusion and intratumor *CTNNB1* expression was only possible due to the inclusion of regions both in and surrounding the tumor. The use of tumor markers (pan-cytokeratin and NY-ESO-1) and a single immune marker (CD45) enabled the selection of ROIs with both immune-rich and immune-poor morphology. The primary and recurrence samples were core needle biopsies and were biased toward the inner tumor bed region, limiting query of the tumor periphery/invasive margin, while the metastasis was a wedge resection, providing a wider margin of the tumor sample. Thus, we may not have had the visibility of the tumor periphery in the primary and recurrence lesions to more specifically identify the patterns of T-cell exclusion. However, all three lesions showed high tumor purity (88%–95%) at the genomic level, suggesting that the gene expression signal in bulk RNA profiling was strongly driven by the tumor cells.

While our approach was successful in identifying immune cell exclusion in the metastatic lesion, we did not identify a clear mechanism of resistance in the recurrence lesion. Genomic analysis did not reveal relevant de novo somatic alterations responsible for disease progression at the primary site. Bulk RNA analysis revealed slight increases in expression of some B cell-related pathways in both the metastasis and recurrence samples, and overall decreases in AP-1 target genes only in the recurrence sample, which has been previously implicated in CAR-T exhaustion.^26^ The gene and protein panels used for spatial profiling did not include AP-1 target genes, preventing us from further interrogating this mechanism, but T-cell exhaustion markers were expressed in regions queried in both the primary and recurrence samples. Spatial profiling did not further illuminate the mechanism of recurrence, since ROIs contained expression of tumor cell marker NY-ESO-1 with other oncogenic pathway markers (eg, *PTEN*, *AKT1*, and *CCND1*) as well as immune lineage and T-cell exhaustion markers. ROIs, which were up to 800 um in diameter, did not provide single-cell resolution in order to describe the phenotype of T cells interacting with tumor cells, emphasizing the importance in selection of imaging platform, ROIs, and markers in using a spatial profiling approach to further study these samples.

This study reports the genomic, transcriptomic, and spatial profiling of an individual patient who demonstrated both metastatic disease progression and primary recurrence following initial regression in response to NY-ESO-1-targeting ACT. While we report these findings in a single patient, which will require future validation in larger cohorts, our approach highlights the advantages in integrating multiple high-throughput molecular profiling techniques in order to characterize individuals or small cohorts with outlier clinical phenotypes, such as ACT resistance.

## Conclusion

Our study used bulk and spatial profiling of the primary and progressive lesions to explore the tumor-immune dynamics responsible for disease progression events following NY-ESO-1-restricted ACT therapy in synovial sarcoma. We identified mutually exclusive expression of *CTNNB1* and immune and T-cell markers in the tumor invasive margin, a mechanism previously described in other tumor types and immunotherapeutic settings. The complementary use of these techniques provide a more highly resolved interrogation of individual case studies, particularly to address complex questions regarding immunotherapeutic mechanisms that require knowledge of the tumor spatial landscape.

## Data Availability

Data are available in a public, open access repository. All data relevant to the study are included in the article or uploaded as supplemental information. All sequencing data are deposited in the Database of Genotypes and Phenotypes and are available through dbGaP accession code phs002762.v1.p1.
